# ENet-6mA: Identification of 6mA Modification Sites in Plant Genomes Using ElasticNet and Neural Networks

**DOI:** 10.3390/ijms23158314

**Published:** 2022-07-27

**Authors:** Zeeshan Abbas, Hilal Tayara, Kil To Chong

**Affiliations:** 1 Department of Electronics and Information Engineering, Jeonbuk National University, Jeonju 54896, Korea; zabbas@jbnu.ac.kr; 2 Institute of Avionics and Aeronautics (IAA), Air University, Islamabad 44000, Pakistan; 3 School of International Engineering and Science, Jeonbuk National University, Jeonju 54896, Korea; 4 Advances Electronics and Information Research Center, Jeonbuk National University, Jeonju 54896, Korea

**Keywords:** bioinformatics, DNA methylation, ElasticNet, epigenomics, epigenome engineering, neural networks

## Abstract

N6-methyladenine (6mA) has been recognized as a key epigenetic alteration that affects a variety of biological activities. Precise prediction of 6mA modification sites is essential for understanding the logical consistency of biological activity. There are various experimental methods for identifying 6mA modification sites, but in silico prediction has emerged as a potential option due to the very high cost and labor-intensive nature of experimental procedures. Taking this into consideration, developing an efficient and accurate model for identifying N6-methyladenine is one of the top objectives in the field of bioinformatics. Therefore, we have created an in silico model for the classification of 6mA modifications in plant genomes. ENet-6mA uses three encoding methods, including one-hot, nucleotide chemical properties (NCP), and electron–ion interaction potential (EIIP), which are concatenated and fed as input to ElasticNet for feature reduction, and then the optimized features are given directly to the neural network to get classified. We used a benchmark dataset of rice for five-fold cross-validation testing and three other datasets from plant genomes for cross-species testing purposes. The results show that the model can predict the N6-methyladenine sites very well, even cross-species. Additionally, we separated the datasets into different ratios and calculated the performance using the area under the precision–recall curve (AUPRC), achieving 0.81, 0.79, and 0.50 with 1:10 (positive:negative) samples for *F. vesca*, *R. chinensis*, and *A. thaliana*, respectively.

## 1. Introduction

DNA N6-methyladenine (6mA), which has been observed in bacteria, eukaryotes, and archaea, is a significant DNA epigenetic alteration [1,2,3]. It is an extensively studied topic in epigenetics and corresponds to methylation at the 6th position of an adenine ring, and has been linked to a variety of biological processes [4,5,6]. Since it had not been detected in previous investigations, DNA 6mA alteration was assumed to be present in prokaryotic genomes only and was not expected to be prevalent in multicellular eukaryotes [7,8]. Due to the advancement of extremely sensitive tools, some studies were able to discover and study the distribution of 6mA in eukaryotic genomes of *Chlamydomonas reinhardtii* [9], *Drosophila melanogaster* [10], and *Caenorhabditis elegans* [11]. Following that, 6mA was examined in *Mus musculus*, *Arabidopsis thaliana*, *rice*, *Xenopus laevis*, and *Danio rerio* [12,13,14,15,16]. These studies revealed that 6mA is widely distributed in eukaryotes and plays an important role in gene regulation.

Experimental techniques have recently been developed to facilitate the exploration of DNA 6mA modifications. Pormraning et al. [17] established a method for analyzing genome-wide DNA methylation in eukaryotes utilizing bisulfite sequencing and methyl-DNA immunoprecipitation. Using laser-induced fluorescence and capillary electrophoresis, Krais et al. [18] demonstrated a quick and sensitive approach for quantifying universal adenine methylation in DNA. Greer et al. [11] examined DNA 6mA levels in *Caenorhabditis elegans* using high throughput liquid chromatography and spectroscopy.

Due to the high cost of experimental techniques, researchers are preferring the in silico prediction methods because of their low cost, time saving, and human error reduction characteristics. The 6mA modification has long been a popular topic in bioinformatics research, and many researchers in this field are now employing machine and deep learning techniques to identify 6mA locations in different genomes. Chen et al. [19] produced a benchmark dataset for 6mA prediction in the rice genome containing 880 positive sequences (6mA) and the same number of negative sequences (non-6mA), and developed an SVM-based tool, i6mA-Pred, to locate 6mA sites in the rice genome, achieving an accuracy of 83%. Pian et al. [20] built a model based on the Markov model and named it MM-6mAPred. They evaluated it using the same benchmark dataset used by i6mA-Pred and outperformed the latter in the prediction of 6mA sites. Using the same dataset, Tahir et al. [21] developed a tool named iDNA6mA. After training and evaluating their model, they reported that iDNA6mA surpassed i6mA-Pred in prediction performance.

FastFeatGen [22] is another tool for predicting 6mA sites that uses a parallel feature extraction technique to collect the most significant features, which are passed into the extra-tree classifier (ETC) for classification. Wahab et al. [23] published a convolutional neural network (CNN)-based model called iIM-CNN, to identify 6mA modifications in various species. Following that, Rehman et al. [4] proposed a CNN and long short-term memory (LSTM)-based method named DNA6mA-MINT, outperforming ilM-CNN. Lv et al. [24] introduced a new benchmark dataset for the prediction of 6mA modifications in the rice genome, containing 154,000 methylated and 154,000 unmethylated sequences. They also proposed a model, iDNA6mA-rice, and evaluated the model using this dataset. They observed good performance. To increase the predictive performance, Haitao et al. [1] proposed a simple but high-performing technique, SNNRice6mA, using one-hot encoding to encode the sequences and CNN as a network, and achieved better performance as compared to iDNA6mA-rice. SpineNet-6mA [2] also proposed a CNN-based architecture using SpinalNet architecture for the first time for sequential data and evaluated the model using the datasets proposed by Chen et al. [19] and Lv et al. [24], outperforming the previous state-of-the-art methods. They also evaluated their model using cross-species testing using *Rosa chinensis* [25] and *Fragaria vesca* [26], achieving better accuracies compared to previous techniques.

All the algorithms discussed above were created with the presumption of equal numbers of positive and negative samples. The data distribution in a real-life scenario can range from a little skewed to severely imbalance, especially in medical cases. This may result in poor prediction accuracy, particularly for the minority class. In this paper, we proposed a tool for the prediction of methylation sites in plant genomes. Unlike traditional models, we tested our model on cross-species imbalanced datasets by dividing the datasets into different ratios and generated the precision–recall curves (PRC) to evaluate the model’s generalizability.

## 2. Results

To prove the superiority of our model over other state-of-the-art models, SNNRice6mA [1], DNA6mA-MINT [4], and SpineNet-6mA [2], we evaluated our model using five-fold cross-validation on the benchmark rice-Lv dataset. We applied the same validation technique to validate ENet-6mA by adopting the same number of folds to obtain a better comparison. To stay in line with the evaluation metrics, we employed the same metrics, including accuracy, sensitivity, specificity, MCC, and AUC. The results of SNNRice6mA and SpineNet-6mA have been directly quoted from the papers, and for DNA6mA-MINT, we trained the whole model again and quote the results in Table 1. In all assessment metrics, our model surpassed the previous state-of-the-art models.

To check whether the model could effectively identify the methylation sites on unseen data and to evaluate its validity, we performed cross-species testing using other similar plant species datasets, including *F. vesca*, *R. chinensis*, and *A. thaliana*. SNNRice-6mA provided their code through GitHub, including the weight files; thus, we ran the testing on their model. Table 2 provides a good comparison among the accuracies and MCCs between our model and SNNRice6mA, DNA6mA-MINT, and SpineNet-6mA. In terms of accuracies, ENet-6mA surpassed SNNRice-6mA by 2.33%, DNA6mA-MINT by 1.79%, and SpineNet-6mA by 0.06%. Therefore, the achieved results show that ENet-6mA outperformed the current state-of-the-art models and thus is a viable resource in computational biology.

In real-world datasets, especially in medical-related datasets, “normal” samples make up the majority of the data—with there being only a tiny fraction of “abnormal” samples—resulting in class imbalance. As a result, algorithms are often swamped by bigger classes and overlook minor classes. Considering this scenario, we divided the datasets into different ratios to test the model on imbalanced datasets. Usually, the number of positive sequences is always low compared to the number of negative sequences; therefore, we divided the datasets into 1:5 and 1:10 (positive:negative) ratios.

In such situations where one truly cares about identifying positive cases, the area under the precision–recall curve (AUPRC) is a valuable performance parameter for imbalanced data. Therefore, we used AUPRC as an evaluation metric for evaluating the imbalanced datasets. Interpreting AUPRC is a little trickier than interpreting the area under the receiver operating characteristic (AUROC), because the benchmark for AUROC is always 0.5, whereas the benchmark for AUPRC is equal to the fraction of positives, where the fraction of positives is determined as: (number of positive sequences/total sequences) [27]. The AUPRC threshold thereby varies for distinct datasets. For a dataset with 15% positive sequences, the benchmark AUPRC is 0.15, so achieving an AUPRC of 0.50 is outstanding. On the other hand, a dataset with 95% positives has a baseline AUPRC of 0.95; therefore, achieving an AUPRC of 0.50 is poor in this case.

**Figure 1:**
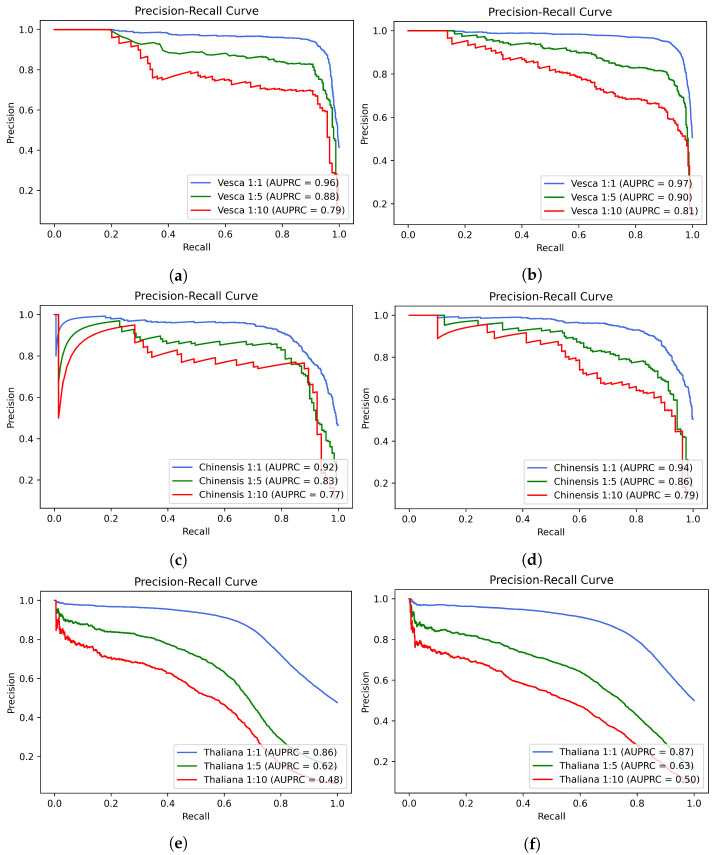
Comparison of the AUPRCs generated with and without ElasticNet on imbalanced datasets of *F. vesca*, *R. chinensis*, and *A. thaliana*. (**a**) *F. vesca* (without ElasticNet); (**b**) *F. vesca* (with ElasticNet); (**c**) *R. chinensis* (without ElasticNet); (**d**) *R. chinensis* (with ElasticNet); (**e**) *A. thaliana* (without ElasticNet); (**f**) *A. thaliana* (with ElasticNet).

Figure 1 shows the precision–recall curves (PRC) generated by the proposed model, ENet-6mA, on *F. vesca*, *R. chinensis*, and *A. thaliana* datasets in 1:1, 1:5, and 1:10 ratios. Total sequences in *F. vesca* are 3932, so 1:5 gives (393 positives:1966 negatives) and 1:10 (196 positives:1966 negatives). In *R. chinensis* we have 1626 sequences, so for 1:5, 162 positives:813 negatives, and for 1:10, (81 positives:813 negatives); and in *A. thaliana* we have 63,746 sequences, so for 1:5, 6374 positives:31,873 negatives, and for 1:10, 3187 positives:31,873 negatives.

## 3. Discussion

### 3.1. Proposed Methodology

**Figure 2:**
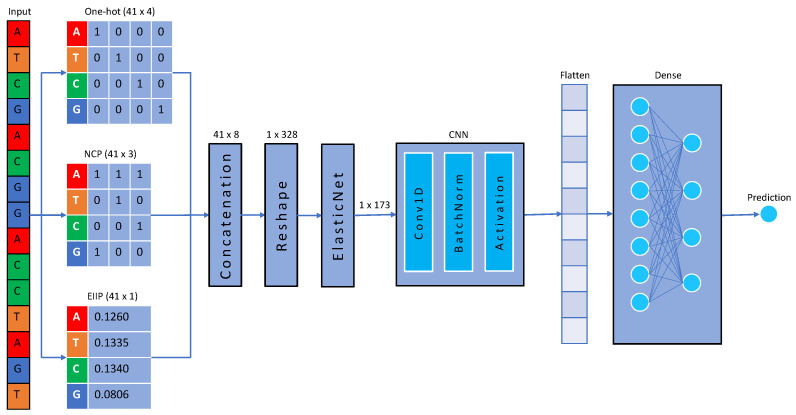
Framework of the proposed model, ENet-6mA.

Every 41 × 8 matrix achieved by concatenating the encoded sequences using one-hot, NCP, and EIIP is reshaped to convert into a row matrix of shape 1 × 328. After converting them into a row matrix, we applied ElasticNet to them to select the best features only. ElasticNet reduced the number of features from 1 × 328 to 1 × 173 for each sequence. To avoid any data leakage, this step was applied to the training data only, and the indexes of reduced features were used to reduce the number of features in the test set. These reduced features were given as input to the CNN for further feature extraction and classification. Figure 2 depicts the overall method for creating the model, ENet-6mA.

**Figure 3:**
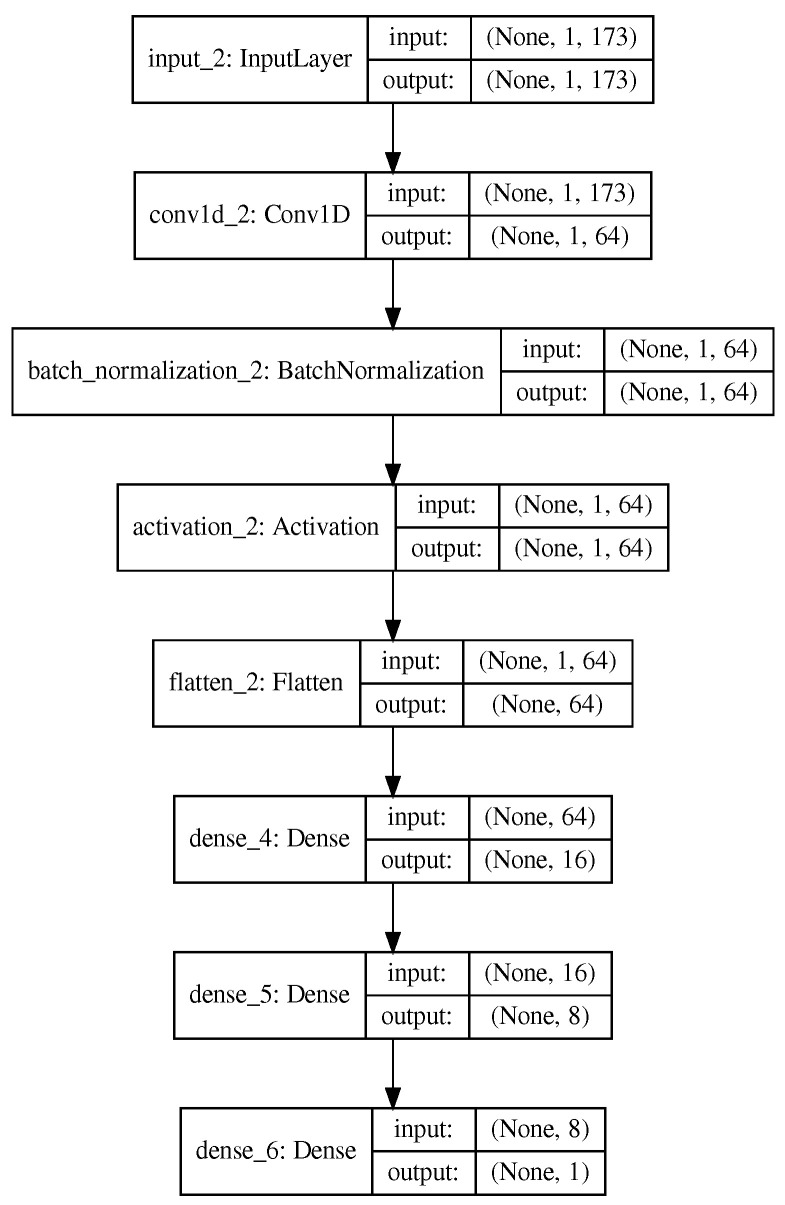
CNN network architecture.

We built a simple CNN-based model using a single convolutional layer containing 64 filters with a kernel size of 7 and a stride of 1. This convolution layer is followed by batch normalization and an activation layer. For batch normalization, we used a momentum of 0.8 for the moving average and an epsilon value of 1 $\times \;10^{-5}$ to avoid *∞* values. The network summary can be seen in Figure 3.

As a non-linear activation function, elu [28] has been used which can be expressed mathematically as:

(1) $$f\left(x\right)=\left\{\begin{array}{ccc} \alpha (e^{x}-1) & for & x\leq 0\\ x & for & x>0 \end{array}\right.$$

After applying the non-linear activation function, we flattened it and fed it as input into the first dense layer with 16 neurons, followed by another dense layer having eight neurons. The final output layer contains only one neuron with a sigmoid activation function, mathematically expressed as:

(2) $$\sigma =\frac{1}{1+e^{-x}}.$$

The sigmoid function [29] returns a float value between 0 and 1, which represents the probability of finding the 6mA change site in the given DNA sequence. The model classifies the sequence as 6mA if the value is more than 0.5, and as non-6mA if the value is less than 0.5. As an optimizer, we employed stochastic gradient descent (SGD) with a momentum of 0.9 and a learning rate of 0.001. Furthermore, we employed early stopping with 10-epoch patience, which stipulated that the training process was to be terminated when the prediction accuracy on the validation set stopped improving for 10 epochs.

### 3.2. Evaluation Metrics

To ensure compatibility with the previous approaches, we used the same five-fold cross-validation technique to evaluate our model. Multiple metrics, including accuracy (Acc), sensitivity (Sn), specificity (Sp), Matthews correlation coefficient (MCC), and area under the curve (AUC), were used to analyze the performance. They can be defined mathematically as:

(3) $$\mathrm{Accuracy}=\mathrm{Acc}=\frac{\mathrm{TP}+\mathrm{TN}}{\mathrm{TP}+\mathrm{TN}+\mathrm{FP}+\mathrm{FN}}$$

(4) $$\mathrm{Sensitivity}=\mathrm{Sn}=\frac{\mathrm{TP}}{\mathrm{TP}+\mathrm{FN}}$$

(5) $$\mathrm{Specificity}=\mathrm{Sp}=\frac{\mathrm{TN}}{\mathrm{TN}+\mathrm{FP}}$$

(6) $$\mathrm{MCC}=\frac{\mathrm{TP}\times \mathrm{TN}-\mathrm{FP}\times \mathrm{FN}}{\sqrt{\left(\mathrm{TP}+\mathrm{FP})(\mathrm{TP}+\mathrm{FN})(\mathrm{TN}+\mathrm{FP})(\mathrm{TN}+\mathrm{FN}\right)}}$$

In addition, we used the area under the precision–recall curve (AUPRC) for imbalance class datasets.

## 4. Materials and Methods

### 4.1. Benchmark Dataset

In this study, the same benchmark rice dataset created by Lv et al. [24] was used for training and five-fold cross-validation testing of the model. The dataset contains 154,000 methylated sequences and the same number of non-methylated sequences. We denote this dataset as rice-Lv in this paper. We also considered three other datasets, *Rosa chinensis*, *Fragaria vesca*, and *Arabidopsis thaliana*, for cross-species testing. For the creation of imbalance datasets, we divided the datasets into different ratios (1:5 and 1:10), where every sequence is 41 base pairs (bp) long. Table 3 shows a comprehensive view of the datasets used in this study.

### 4.2. Data Representation

The sequences in the dataset are in string format, as in “ATCTAGG…CGAATTA”, which is not readable by a machine. Therefore, we needed to convert them first into a machine-readable format. To make them machine-readable, we have used three different encoding techniques, including one-hot, nucleotide chemical properties (NCP), and electron–ion interaction potential (EIIP).

### 4.3. One-Hot-Encoding

One-hot-encoding is a simple and efficient way to transform nucleotides into a machine-readable format. It is one of the widely used encoding schemes in the field of bioinformatics [30,31,32]. Using this encoding method, the four nucleotides, adenine (A), thymine (T), cytosine (C), and guanine (G), can be represented as A: 1,0,0,0; T: 0,1,0,0 C: 0,0,1,0, and G: 0,0,0,1, respectively. Thus, for each sequence of length L, every sequence S can be represented as an S×4 dimensional vector.

### 4.4. Nucleotide Chemical Properties

Nucleotide chemical properties (NCP) is a depiction of each nucleotide in a three-dimensional Cartesian coordinate system based on three chemical groups [33]. Considering the ring structure, adenine and guanine consist of two rings each and are called purines, whereas cytosine and uracil have only a single ring and are called pyrimidines. While building secondary structures, A and U share a weak bond, whereas C and G share a strong bond. In terms of chemical functionality, A and C belong to the amino group, whereas G and U belong to the keto group. Therefore, the nucleotides can be classified in the three-dimensional coordinate system into three different groups based on these chemical properties. The nucleotide A can be represented as (1,1,1), T as (0,1,0), C as (0,0,1), and G as (1,0,0).

### 4.5. Electron–Ion Interaction Potential

Electron–ion interaction potential (EIIP)-based classification [34] has been widely used to solve a variety of prediction issues with utmost precision. Using this encoding scheme, we can assign values for each nucleotide as A: 0.1260, T: 0.1335, C: 0.1340, and G: 0.0806.

As each sequence was of length 41, one-hot-encoding resulted in a matrix of 41 × 4, NCP resulted in 41 × 3, and EIIP created a matrix of 41 × 1. After applying these encoding techniques, we concatenated them to create a matrix of 41 × 8 for each sequence.

### 4.6. Elastic Net

Elastic net is a linear regression model proposed by Zou et al. [35], based on ridge and Lasso regression techniques, and has already been used by many researchers [36,37,38]. Given that the ridge regression is susceptible to distortion while Lasso is oversimplified, elastic net evolved to overcome the constraints of the two approaches. It can be defined as:

(7) $${\hat{\beta }}_{\mathrm{enet}}=\left(1+\frac{\lambda_{2}}{n}\right)\left\{\arg\min_{\beta }{∥y-\sum_{j=1}^{P}x_{j}\beta_{j}∥}^{2}+\lambda_{1}{\left\|\beta \right\|}_{1}+\lambda_{2}{\left\|\beta \right\|}_{2}^{2}\right\}$$

where β is the regression coefficient, and $\lambda_{1}$ and $\lambda_{2}$ are tuning parameters (always positive). In this equation, if we put $\lambda_{1}$ = 0, it gives ridge regression, whereas $\lambda_{2}$ = 0 will result in Lasso regression. Elastic net can be considered as a penalized least square method. The penalty term $\lambda_{1}{\left\|\beta \right\|}_{1}+\lambda_{2}{\left\|\beta \right\|}_{2}^{2}$ is the convex summation of ridge and Lasso penalties.

## 5. Conclusions

In this study, we proposed a model based on ElasticNet and neural networks for the identification of DNA N6-methyladenine (6mA) sites in plant genomes. To extract the unique characteristics of the sequences, we used three encoding schemes: one-hot, NCP, and EIIP. The encoded sequences were then concatenated and given as input to the ElasticNet to remove the unnecessary features. After the reduction of features using ElasticNet, we applied neural networks for the classification of sequences as either methylated or not. ENet-6mA got almost the same results as the previous model while testing 5-fold cross-validation, but performed much better than all the previous models when tested on cross-species and imbalanced datasets. Since we trained the model on one species and tested it on other plant species, we anticipate that our model can facilitate the identification of methylation sites in different plant species. A user-friendly webserver has been made publicly available at our project website.

## Figures and Tables

**Table 1:** Performance comparison of ENet-6mA with other state-of-the-art methods using five-fold cross-validation testing.

Methods	Acc	Sn	Sp	MCC	AUC
SNNRice6mA	0.9204	0.9433	0.8975	0.84	0.97
DNA6mA-MINT	0.9258	0.9012	0.9306	0.85	0.97
SpineNet-6mA	0.9431	0.9571	0.9292	0.88	0.98
Proposed	0.9437±0.0003	0.9467±0.002	0.9339±0.002	0.87±0.0008	0.98±0.0007

**Table 2:** Cross-species performance comparison between ENet-6mA and other state-of-the-art-models on *R. chinensis*, *F. vesca*, and *A. thaliana* datasets.

Methods	*R. chinensis*		*F. vesca*		*A. thaliana*	
	Accuracy (%)	MCC	Accuracy (%)	MCC	Accuracy (%)	MCC
SNNRice6mA-large	81.13	0.62	87.84	0.75	77.6	0.57
DNA6mA-MINT	82.43	0.64	88.11	0.76	76.21	0.56
SpineNet-6mA	85.20	0.70	90.30	0.80	76.15	0.56
Proposed	87.75	0.75	93.20	0.86	79.14	0.60

**Table 3:** Benchmark dataset; *Rice-Lv*; and cross-species testing datasets, *Fragaria vesca*, *Rosa chinensis*, and *Arabidopsis thaliana*, used in this study.

Dataset	Pos Samples	Neg Samples	Total	Family
*Rice-Lv*	154,000	154,000	308,000	rice
*F. vesca*	1966	1966	3932	rosaceae
*R. chinensis*	813	813	1626	rosaceae
*A. thaliana*	31,873	31,873	63,746	brassicaceae

## Data Availability

A user-friendly webserver has been made publicly available at, http://nsclbio.jbnu.ac.kr/tools/ENet-6mA/, accessed on 23 July 2022, and the datasets used along with the source code is made available for researchers via GitHub at https://github.com/Z-Abbas/ENet-6mA, accessed on 23 July 2022.
